# Rebuilding life after heart valve surgery: The VALCAR(E)_QoL study on rehabilitation and quality of life

**DOI:** 10.1016/j.mex.2025.103781

**Published:** 2025-12-25

**Authors:** Piergiuseppe Liuzzi, Camilla Elena Magi, Paolo Iovino, Ercole Vellone, Andrea Mannini, Claudio Macchi, Biagio Nicolosi, Mariachiara Figura, Francesco Limonti, Duccio Frangi, Hamilton Dollaku

**Affiliations:** a IRCCS Fondazione Don Carlo Gnocchi ONLUS, Florence, Italy; b Health Sciences Department, University of Florence, Florence, Italy; c Department of Biomedicine and Prevention, University of Rome Tor Vergata, Rome, Italy; d Department of Nursing, Faculty of Nursing and Midwifery, Wroclaw Medical University, Wroclaw, Poland; e Department of Health Promotion, Maternal and Infant Care, Internal Medicine and Medical Specialties (PROMISE), University of Palermo, Palermo, Italy

**Keywords:** Cardiac rehabilitation, Health-related quality of life, Patient-reported outcomes, Psychosocial recovery, Self-care and self-efficacy, Functional capacity, International Classification of Functioning, Disability and Health (ICF), Post-surgical recovery, Predictors of rehabilitation outcomes

## Abstract

The VALCAR(E)_QoL study is a prospective, mixed-methods observational investigation designed to characterize the rehabilitation process and determinants of health-related quality of life in patients undergoing cardiac valve surgery. Conducted at the IRCCS Fondazione Don Carlo Gnocchi in Florence (Italy), the study integrates quantitative and qualitative approaches to capture the multidimensional nature of recovery within the International Classification of Functioning, Disability and Health (ICF) framework.

Consecutive patients admitted for inpatient cardiac rehabilitation after valve surgery will be assessed at four time points: pre-surgery (T0), rehabilitation admission (T1), discharge (T2), and six-month follow-up (T3). Data collection includes clinical, functional, and psychosocial indicators, as well as patient-reported outcomes such as the Kansas City Cardiomyopathy Questionnaire (primary outcome), SF-12 Health Survey, and standardized measures of anxiety, depression, stress, self-care, and sleep quality. Qualitative interviews at discharge complement quantitative findings by exploring patients’ emotional experiences, perceived barriers, and facilitators of recovery.

All data are collected in pseudonymized form through a secure REDCap database and analyzed using multivariate and thematic techniques. This protocol adheres to the STROBE guidelines to ensure methodological transparency and reproducibility.

By integrating objective measures and subjective experiences, the VALCAR(E)_QoL study aims to identify clinical and psychosocial predictors of successful rehabilitation and to provide evidence for patient-centered, multidisciplinary models of post-surgical care for individuals recovering from heart valve surgery.

## Graphical abstract

**Figure:**
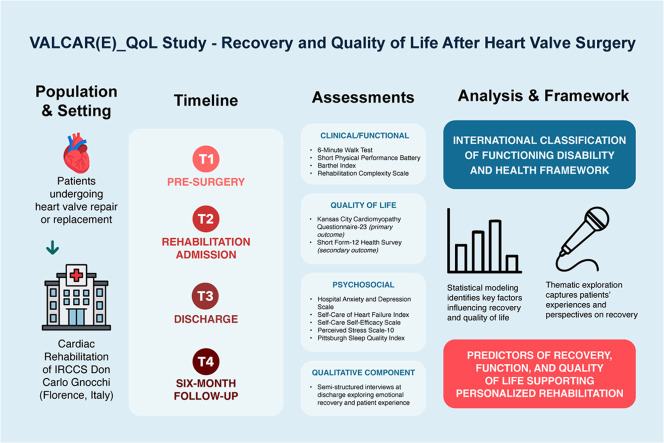

**Related research article**

*None*

***For a published article:***

**None**

**Specifications table**

**Table:** 

**Subject area**	Medicine and Dentistry
**More specific subject area**	*Cardiac Rehabilitation, Patient-Centered Outcomes*
**Name of your method**	*Protocol of a prospective mixed-methods observational study*
**Name and reference of original method**	*N/A*
**Resource availability**	*N/A*

## Background

Valvular heart disease is a cardiac disorder resulting from structural or functional alterations of the heart valves, leading to hemodynamic compromise, progressive cardiac dysfunction, and a reduced quality of life [1]. Its prevalence is approximately 2–3 % in the general population and increases to nearly 13 % among individuals over 75 years of age [2]. Aortic stenosis and mitral regurgitation are the most common forms, primarily related to degenerative, rheumatic, or congenital etiologies. In high-income countries, calcific aortic stenosis predominates, driven by age and cardiovascular risk factors such as hypertension and hyperlipidemia [3]. The global burden of valvular heart disease continues to rise with population aging, with a sevenfold increase in calcific aortic valve disease over the past three decades [1,4].

Surgical or transcatheter valve interventions represent the standard of care for advanced valvular heart disease, with >250,000 procedures performed annually in Western countries [5]. Although surgery significantly improves survival, postoperative recovery remains challenging, with many patients experiencing reduced functional capacity, emotional distress, and difficulties in readapting to daily life [[6], [7], [8]]. Therefore, optimizing rehabilitation and long-term recovery in these patients is essential.

Cardiac rehabilitation (CR) is a structured, multidisciplinary intervention endorsed by international guidelines [9,10] Evidence demonstrates that exercise-based CR improves physical capacity and self-care following valve surgery [[11], [12], [13], [14]]. However, participation remains low, with fewer than half of eligible patients attending CR programs [15,16]. Barriers such as advanced age, frailty, and limited referral pathways contribute to this underuse [17]. Nurses play a pivotal role throughout the rehabilitation process, promoting patient education, symptom management, and psychosocial adjustment as part of the multidisciplinary “heart team” [[18], [19], [20]]. Innovative models, including home-based and tele-rehabilitation have demonstrated comparable outcomes to traditional programs—particularly among older adults [21,22] - while peer-support interventions can improve adherence and reduce perioperative anxiety [23].

However, current evidence on rehabilitation outcomes after valve surgery remains limited. Most studies are small, focus mainly on exercise capacity, and rarely investigate patient-reported outcomes such as quality of life, emotional well-being, or self-care behaviors [8,[24], [25], [26]]. Only a few qualitative studies have explored patients’ lived experiences and psychosocial recovery [[27], [28], [29]]. These gaps hinder the development of patient-centered rehabilitation pathways tailored to the specific needs of this population.

This study is grounded in the World Health Organization’s *International Classification of Functioning, Disability and Health* framework, which conceptualizes recovery as the result of dynamic interactions among physical, psychological, and social dimensions of health [30]. According to this perspective, quality of life after cardiac valve surgery depends not only on functional restoration but also on the individuals’ ability to resume participation in everyday and social activities.

## Method details

The VALCAR(E)_QoL study will be a prospective observational investigation conducted at the IRCCS Fondazione Don Carlo Gnocchi in Florence, Italy. Its primary aim will be to evaluate the rehabilitation process and quality of life in patients undergoing cardiac valve surgery. The study will employ a mixed-methods design, integrating quantitative assessments with qualitative evaluations to provide a comprehensive understanding of patients’ recovery trajectories.

Data collection will take place during the inpatient cardiac rehabilitation phase and at a six-month follow-up after hospital discharge. Participants will be consecutively recruited during their hospitalization in the Cardiac Rehabilitation Unit between September 2025 and September 2027. All clinical and patient-reported data will be collected as part of routine care and entered in a pseudonymized, secure electronic database using the REDCap (Research Electronic Data Capture) platform [31]. The study protocol adheres to the *Strengthening the Reporting of Observational Studies in Epidemiology* (STROBE) guidelines [32] to ensure transparency, methodological rigor, and high-quality reporting.

### Aim

The VALCAR(E)_QoL study will aim to evaluate the clinical, functional, and psychological factors associated with quality of life at discharge from cardiac rehabilitation (T2) in patients who have undergone cardiac valve surgery. The primary endpoint will be the *Kansas City Cardiomyopathy Questionnaire-23* (KCCQ-23) score at discharge, which assesses patients’ health-related quality of life. Secondary objectives will include identifying predictors of successful rehabilitation, defined by improvements in functional capacity, self-care ability, and psychosocial adjustment, and assessing changes in anxiety, depression, and sleep quality during and after rehabilitation. The study is conceptually grounded in the *International Classification of Functioning, Disability and Health* framework, which provides a multidimensional perspective integrating physical, psychological, and social aspects of recovery [30]. A qualitative component will complement the quantitative analyses through semi-structured interviews conducted at discharge, designed to explore patients’ experiences, emotional responses, and perceptions of their recovery process.

### Expected results

This study is expected to provide a comprehensive characterization of the rehabilitation trajectory of patients undergoing cardiac valve surgery. By integrating quantitative and qualitative data, we aim to identify patterns of physical, functional, and psychosocial recovery during the rehabilitation period and at six months after discharge. We hypothesize that specific clinical and psychosocial characteristics at admission, such as age, baseline frailty, comorbidities, self-care ability, and emotional status, will be associated with varying degrees of improvement in functional independence and quality of life over time. The qualitative findings are expected to complement these analyses by elucidating patients’ perceived needs, challenges, and facilitators during recovery, thereby helping to define the specific rehabilitation priorities of this population. Consistent with the *International Classification of Functioning, Disability and Health* framework, results will be interpreted by considering how body functions, activities, participation, and contextual factors interact to shape recovery and quality of life. Rather than testing the efficacy of rehabilitation, the study focuses on observing and modeling these multidimensional associations to inform more individualized and patient-centered rehabilitation pathways for individuals undergoing valve surgery.

### Setting

This study will be conducted at the Cardiac Rehabilitation Unit of the IRCCS Fondazione Don Gnocchi in Florence, Italy. Consecutive patients meeting the inclusion criteria will be enrolled between September 2025 and September 2027. All participants will follow the standard clinical care pathway routinely implemented in the center. Within this framework, each patient will receive an individualized rehabilitation program (*Progetto Riabilitativo Individuale*, PRI) developed by the multidisciplinary team according to the patient’s clinical condition and specific recovery goals. Rehabilitation activities will be performed both in the inpatient ward and in the institute’s dedicated facilities, including the gym and therapy rooms.

### Population

The study population will include patients who will have undergone cardiac valve surgery and are admitted to the Cardiac Rehabilitation Unit of the IRCCS Fondazione Don Gnocchi in Florence for postoperative rehabilitation. These patients will typically be in a phase of transient and potentially reversible clinical instability following cardiac surgery and undergo a structured recovery program aimed at restoring functional capacity, autonomy, and self-care abilities. The rehabilitation process will be designed to support the gradual transition toward clinical stability and improved quality of life, integrating physical reconditioning with educational and psychosocial interventions as part of routine care.

### Eligibility criteria

#### Inclusion criteria

Participants will be adults aged 18 years or older who have undergone heart valve surgery, including any type of valvular repair or replacement, either as isolated procedures or in combination with other cardiac interventions. All participants must have sufficient cognitive ability to engage in the rehabilitation program and to provide informed consent, as verified by a Six-Item Screener (SIS) score of ≥4.

#### Exclusion criteria

Patients will be excluded if they are unable to participate in the rehabilitation program due to severe cognitive, psychiatric, or neurological conditions (e.g., advanced dementia or delirium) that prevent them from following instructions. Individuals who refuse or withdraw informed consent will also be excluded from participation.

#### Patient registration

Patients will be prospectively enrolled in the Cardiac Rehabilitation Unit of the IRCCS Fondazione Don Carlo Gnocchi in Florence. Eligible participants will be identified during their hospital stay after cardiac valve surgery and are consecutively recruited between September 2025 and September 2027. All eligible patients will be informed about the study objectives and procedures during hospital admission, and written informed consent will be obtained prior to participation. Data collection will begin at admission and will continue throughout the inpatient rehabilitation period, with a scheduled follow-up assessment six months after discharge, conducted either in person or by telephone, depending on patient availability.

#### Data collection

The VALCAR(E)_QoL study will be an observational investigation embedded in routine clinical practice, collecting standardized rehabilitation data without introducing additional procedures or experimental interventions. This design will ensure ecological validity and accurately reflects real-world rehabilitation processes. Pseudonymized data will be collected prospectively from all patients enrolled in the study during their hospitalization at the Cardiac Rehabilitation Unit of the IRCCS Fondazione Don Carlo Gnocchi in Florence and at a scheduled six-month follow-up. The data collection process will include a range of clinical, functional, and psychosocial assessments, as well as patient-reported outcome measures, performed at predefined time points. The *International Classification of Functioning, Disability and Health* framework will serve as the interpretative model to understand how physical, psychological, and social dimensions interact during recovery. Table 1 summarizes the psychometric instruments used in the study, while the assessments are performed at the following time points and are consistently grouped by domain.

**Table 1:** Psychometric and standardized instruments used in the VALCAR(E)_QoL study and their main measurement properties.

Instrument	Items	Response format	Score range / Interpretation	Dimension / Subscales	Reliability	Reference
Charlson Comorbidity Index (CCI)	19	Weighted index	0–37; higher = greater comorbidity burden	19 comorbidity categories	–	(Akhtar et al., 2024)
Braden Scale	6	4-point Likert	6–23; lower = higher risk	Sensory perception, moisture, activity, mobility, nutrition, friction/shear	α = 0.83–0.95	(Huang et al., 2021)
PUSH Tool 3.0	3	Ordinal	0–17; lower = better healing	Surface area, exudate amount, tissue type	α = 0.83	(Gardner et al., 2005)
NYHA Classification	4	Clinical rating	I–IV; higher = more severe symptoms	Functional limitation due to heart failure	Inter-rater reliability = 0.90	(Bennett et al., 2002)
MUST	3	Weighted score	0–6; ≥2 = high risk	BMI, unplanned weight loss, acute disease effect	–	(Sharma et al., 2022)
Modified Barthel Index	10	5-point Likert	0–100; higher = more independence	ADL: feeding, mobility, grooming, etc.	α = 0.86–0.92	(Galeotto, 2015)
SPPB	3 tests	Performance-based	0–12; higher = better function	Balance, gait speed, chair stand	α = 0.81–0.88	(Guralnik et al., 1994)
6-Minute Walk Test (6MWT)	–	Meters walked	–; higher = better functional capacity	Aerobic endurance	ICC = 0.94–0.97	(Bellet et al., 2012)
Borg RPE Scale	15	6–20	6–20; higher = greater perceived effort	Perceived exertion	r = 0.80 with HR	(Borg, 2007; Callahan et al., 2002)
Six Item Screener (SIS)	6	Binary	0–6; <4 = cognitive impairment	Orientation, recall	Sensitivity = 88 %	(Callahan et al., 2002)
Rehabilitation Complexity Scale (RCS-E v13)	13	Weighted score	0–50; higher = greater rehabilitation needs	Care, nursing, therapy, medical, equipment	α = 0.82	(Roda et al., 2017)
Self-Care Self-Efficacy Scale (SCSES)	10	5-point Likert (1–5)	0–100; higher = greater self-efficacy	Self-care maintenance, monitoring, management	α = 0.89–0.93; ω ≥ 0.90	(Yu et al., 2021)
Self-Care of Heart Failure Index (SCHFI v8.0)	22	4-point Likert (1–4)	0–100; ≥70 = adequate self-care	Maintenance, Management, Confidence	α = 0.78–0.88; ω = 0.76–0.90	(Vellone et al., 2013)
SF-12 Health Survey	12	3–5-point Likert	PCS/MCS 0–100; higher = better QoL	Physical & Mental Health summary scores	α = 0.80–0.89	(Gandek et al., 1998)
Kansas City Cardiomyopathy Questionnaire – 23 items (KCCQ-23)	23	5-point Likert	0–100; higher = better health status	Physical Limitation, Symptoms, Social Interference, QoL, Self-Efficacy; summary scores: Symptom, Functional, Clinical, Overall	α = 0.89–0.96; ω = 0.77–0.93	[12]
Perceived Stress Scale (PSS-10)	10	5-point Likert (0–4)	0–40; higher = more stress	Perceived helplessness and self-efficacy	α = 0.78–0.91	(Cohen & Janucki-Deverts, 2012)
Hospital Anxiety and Depression Scale (HADS)	14	4-point Likert (0–3)	0–21 per subscale; ≥8 = possible case	Anxiety, Depression	α = 0.83–0.90	(Iani et al., 2014)
Engagement Scale	12	5-point Likert	0–48; higher = greater engagement	Cognitive, emotional, behavioral engagement	α = 0.87–0.92	(Scialpi et al., 2022)
Pittsburgh Sleep Quality Index (PSQI)	19	Mixed 0–3 scale	0–21; >5 = poor sleep quality	7 components (subjective quality, latency, duration, etc.)	α = 0.73–0.83	(Scialpi et al., 2022)

T0: Pre-surgery baseline. Before valve surgery, demographic and clinical characteristics, comorbidities, and preoperative clinical status will be collected. Functional status will be documented using routine clinical assessment. Quality of life will be assessed using the Short Form-12 Health Survey (SF-12) and the Kansas City Cardiomyopathy Questionnaire-23 (KCCQ-23). Psychosocial well-being and sleep will be evaluated using the Hospital Anxiety and Depression Scale (HADS), the Perceived Stress Scale (PSS-10), and the Pittsburgh Sleep Quality Index (PSQI). Self-care and related self-efficacy will be assessed using the Self-Care of Heart Failure Index (SCHFI) and the Self-Care Self-Efficacy Scale (SCSE).

T1: Admission to cardiac rehabilitation. At entry into the rehabilitation unit, clinical variables such as cardiac status, post-surgical complications, medication use, and nutritional and pressure-injury risk will be recorded. Functional performance will be assessed using the 6-Minute Walk Test (6MWT), the Short Physical Performance Battery (SPPB), the Barthel Index, and the Rehabilitation Complexity Scale (RCS). Quality of life will be evaluated using the SF-12 and KCCQ-23. Psychosocial and sleep parameters will be assessed with HADS, PSS-10, and PSQI. Self-care and self-efficacy will be measured using the SCHFI and SCSE.

T2: Discharge from inpatient rehabilitation. At discharge, clinical indicators will include left ventricular ejection fraction, type of surgery, nutritional risk (MUST), pressure-injury risk (Braden Scale), and rehabilitation complexity (RCS). Functional outcomes will be reassessed using the 6MWT, SPPB, and Barthel Index. Quality of life will be measured with the SF-12 and KCCQ-23. Psychosocial distress, perceived stress, and sleep quality will be reassessed using HADS, PSS-10, and PSQI. Self-care abilities and self-efficacy will be again evaluated using the SCHFI and SCSE.

T3: Six-month follow-up. At six months, clinical outcomes will include rehospitalizations and major adverse clinical events. Functional independence will be assessed using the Barthel Index. Quality of life will be evaluated using the SF-12 and KCCQ-23. Psychosocial well-being and sleep will be reassessed with HADS, PSS-10, and PSQI. Self-care behaviors and self-efficacy will be re-evaluated using the SCHFI and SCSE.

In addition, qualitative data will be collected through semi-structured interviews conducted at discharge (T2). These interviews will explore patients’ experiences during hospitalization and rehabilitation, focusing on emotional responses, perceived recovery, and support systems. Interviews will be audio-recorded with informed consent, transcribed verbatim, and analysed to understand how physical, psychological, and social dimensions interact during recovery.

#### Sample size

The exact number of patients to be included in the study will not be determinable a priori, as this represents a convenience sample of all eligible patients over the 24-month recruitment period. Based on historical admission volumes to the rehabilitation unit and estimated eligibility rates, we anticipate enrolling approximately 400–450 patients over two years. All eligible patients will be invited to participate, and those providing informed consent will be included. For planning purposes and to ensure adequate statistical power for the primary objective, we considered existing data on factors influencing quality of life after cardiac interventions. Based on data reported by Osnabrugge et al [33], using the most conservative association observed (odds ratio = 1.9 for a predictor of poor outcome, 95 % CI = 1.2–3.3), we performed an approximate sample size estimation [33]. Assuming a significance level of α = 0.05 and a power (1−β) = 0.95 to detect such an association in a multivariable model, with an outcome prevalence of approximately 24 % and accounting for a moderate R² of 0.5 among covariates, around 373 participants will be required per variable of interest [33]. Considering up to 10 % potential attrition or loss to follow-up at six months, a target of approximately 411 patients will be estimated to ensure sufficient power. Our expected sample size of over 400 patients aligns with this estimation, ensuring adequate precision and allowing robust exploratory analyses of multiple predictors. For the qualitative component, the sample size of interview participants will be determined according to the principle of data saturation. We will continue conducting interviews until we reach saturation, that is, interviews will continue until no new themes or insights emerge. Based on previous qualitative research in similar contexts, we anticipate that 20–30 interviews will be sufficient to achieve thematic saturation. The final number will be justified explicitly in the study report following established standards of transparency and comprehensiveness [34,35].

#### Potential participant risks and benefits

This observational study will be considered low risk. There will be no additional clinical risks to participants beyond those inherent to standard care (i.e., rehabilitation and follow-up). The study will not involve any experimental therapeutic interventions or deviations from routine clinical practice. All physical assessments (e.g., exercise tests) will be part of routine rehabilitation and will be conducted with appropriate medical supervision, thus posing minimal incremental risk. A minor risk of fatigue or discomfort may arise from completing questionnaires or participating in interviews; however, participants may take breaks as needed and may skip any question they prefer not to answer. The risk to privacy violation is minimal and mitigated through strict data de-identification and confidentiality procedures. Although participants may not receive direct personal benefits from participation, some individuals may find discussing their experiences during interviews to be cathartic or empowering, as also evidenced by previous documented experiences [36,37]. Indirectly, participants will contribute to advancing knowledge that could improve rehabilitation services for future patients undergoing cardiac valve surgery. All patients will continue to receive the full standard of care regardless of study participation, and they may withdraw from the study at any time without any consequences for their medical treatment.

### Outcome

#### Primary outcome

The primary outcome of the study will be patients’ health-related quality of life at discharge from inpatient cardiac rehabilitation (T2), measured using the Kansas City Cardiomyopathy Questionnaire-23 (KCCQ-23). Both the Clinical Summary Score (CSS) and the Overall Summary Score (OSS) will be analyzed as continuous variables. Baseline clinical, functional, and psychosocial variables collected at pre-surgery (T0) and at admission to rehabilitation (T1) will be examined as potential predictors of KCCQ-23 scores at discharge (T2). In addition, longitudinal changes in health-related quality of life will be explored by reassessing the KCCQ-23 at the six-month follow-up (T3). The 12-Item Short Form Health Survey (SF-12), providing the Physical and Mental Component Summary scores, will be used as a secondary generic measure of quality of life and administered at T0, T1, T2, and T3, with a specific focus on changes between discharge (T2) and follow-up (T3). Overall, the primary outcome analysis will aim to identify the main determinants of health-related quality of life at discharge and to describe its evolution over time.

#### Secondary outcomes

Secondary outcomes are grouped by domain and assessed across the predefined time points as follows:

• Functional outcomes (T1, T2, T3). Functional status will be assessed at admission to rehabilitation (T1), discharge (T2), and six-month follow-up (T3) using the Barthel Index, Short Physical Performance Battery (SPPB), and 6-Minute Walk Test (6MWT). Changes over time in functional independence, physical performance, and exercise capacity will be analyzed as indicators of rehabilitation effectiveness.
• Self-care abilities and self-efficacy (T0, T1, T2, T3). Self-care behaviors and related self-efficacy will be evaluated longitudinally using the Self-Care of Heart Failure Index (SCHFI v8.0) and the Self-Care Self-Efficacy Scale (SCSE) at all study time points. These outcomes reflect patients’ capacity and confidence in managing symptoms, treatment adherence, and lifestyle behaviors after valve surgery.
• Psychosocial and sleep-related outcomes (T0, T1, T2, T3). Anxiety and depressive symptoms will be assessed using the Hospital Anxiety and Depression Scale (HADS), perceived stress using the Perceived Stress Scale-10 (PSS-10), and sleep quality using the Pittsburgh Sleep Quality Index (PSQI). These instruments will be administered longitudinally from baseline (T0) through follow-up (T3) to capture trajectories of psychological well-being during recovery. Patient emotional and cognitive engagement in the rehabilitation process will be assessed using the Patient Health Engagement Scale from T1 to T3.
• Clinical and physical status outcomes (T0, T1, T2, T3). Key clinical parameters will include left ventricular ejection fraction, type of valve surgery, and post-operative complications will be recorded from baseline through discharge. Frailty, physical resilience, and rehabilitation burden will be evaluated using gait speed and the Rehabilitation Complexity Scale (RCS) at T1 and T2. Comorbidity burden will be assessed at baseline using the Charlson Comorbidity Index (CCI). Nutritional status will be screened using the Malnutrition Universal Screening Tool (MUST), and skin integrity will be monitored using the Braden Scale during the inpatient rehabilitation phase.
• Narrative (qualitative) outcomes (T2). Qualitative outcomes will be derived from semi-structured interviews conducted at discharge (T2) to explore patients’ emotional experiences, perceived challenges, facilitators of recovery, and the perceived impact of rehabilitation on daily life.

### Statistical analysis

#### Descriptive statistics and missing data handling

Quantitative analyses will be performed across the four time points, T0 (pre-surgery), T1 (rehabilitation admission), T2 (rehabilitation discharge), and T3 (six-month follow-up), and tailored to each specific outcome. Continuous variables will be reported as mean ± standard deviation or median (interquartile range, IQR), depending on data distribution (assessed using the Shapiro-Wilk test). Categorical variables will be summarized as counts and percentages. All datasets will be screened for missingness. If <5 % of data are missing, complete case analysis will be used. Otherwise, multiple imputation by chained equations (MICE) will be applied under the assumption of missing at random (MAR). Sensitivity analyses will be performed to compare results from complete-case and imputed datasets. Statistical analysis and multivariate supervised/unsupervised modeling will be performed using IBM SPSS v27 and Python 3 custom code.

#### Primary outcome

##### Endpoint definition and temporal constraints

The primary endpoint is health‑related quality of life at discharge (T2), measured using the KCCQ‑23 CSS and OSS treated as continuous outcomes. The same analytical framework will be applied to KCCQ‑23 at six‑month follow‑up (T3). To preserve temporal ordering, models for T2 include only predictors measured at pre‑surgery (T0) and at rehabilitation admission (T1), whereas models for T3 will be estimated under two specifications: (i) including T0 + T1 predictors only, and (ii) including T0 + T1 + T2 predictors, to quantify the incremental information provided by discharge-level data. Continuous predictors will be mean‑centred and standardized, while categorical predictors will be dummy‑coded.

Step 1 *- Univariate screening (group‑level associations)*

For each endpoint (T2 and T3), candidate predictors will be screened for association with KCCQ‑23 scores using statistical tests matched to variable scale and distribution: Pearson or Spearman correlation coefficients for continuous–continuous pairs; two‑sample t‑tests (Welch‑corrected when variances are unequal) or Mann-Whitney U tests for binary predictors; and one-way ANOVA (Welch) or Kruskal–Wallis tests for multi‑level categorical variables. Effect sizes will be reported as standardized regression coefficients (β), correlation coefficients (r), Hedges’ g, or η²/ε² as appropriate, together with 95 % confidence intervals (CIs). Two‑sided p‑values will be adjusted within conceptual variable domains (clinical, functional, psychosocial/self‑care, treatment‑related) using the Benjamini–Hochberg false‑discovery rate (FDR) correction. Variables meeting *q* < 0.05 (or *p* < 0.05 where FDR is not applicable) will be entered into multivariable modelling.

Step 2 *- Multivariable association models with bootstrap inference*

For T2, all variables retained at Step 1 (from T0 and T1) will enter a multivariable linear regression for KCCQ‑23, estimated with heteroskedasticity‑consistent standard errors. The same procedure will be repeated for T3 using only T0 and T1 variables only. Degrees‑of‑freedom will be controlled a priori (domain-specific caps) to prevent overfitting. Multicollinearity will be monitored (VIF < 5), and highly correlated predictors will be reduced accordingly. Model diagnostics will include checks for linearity, residual structure, and influential observations (Cook’s distance). Bias‑corrected and accelerated (BCa) bootstrap 95 % CIs will be computed for regression coefficients and global fit indices using 1000 resamples. Results will be presented as standardized coefficients (ΔSD in KCCQ‑23 per 1 SD change in predictor), adjusted R², and domain‑level partial R², emphasizing effect sizes and interval estimates.

Step 3 *- Supervised predictive modelling and incremental value of T2 for T3*

Prediction models will target KCCQ‑23 at T2 (features: T0 + T1) and at T3 under two specifications: (i) an early model (T0 + T1) and (ii) an augmented model (T0 + T1 + T2). Preprocessing will follow the missing‑data strategy described above, with imputation and standardization performed within a cross-validation strategy to prevent information leakage. Candidate algorithms will include (a) linear/regularized models (ridge, lasso, and elastic‑net regression) and (b) tree‑based ensembles (random forest and gradient boosting). Hyperparameters will be tuned via nested 10‑fold cross‑validation (inner loop: tuning; outer loop: generalization error) with performance metrics including R², RMSE, and MAE averaged across outer folds. For T3, the incremental predictive value of adding T2 information will be quantified by ΔR², ΔRMSE/ΔMAE, changes in calibration parameters, and paired bootstrap tests on fold‑wise performance differences. Feature importance will be summarized as standardized coefficients for linear/regularized models and as permutation importance for ensembles, complemented by post-hoc, patient-level interpretability methods (e.g., SHAP).

Step 4 *- Patient‑centred analyses: unsupervised T0–T1 endotypes*

**Fig. 1:**
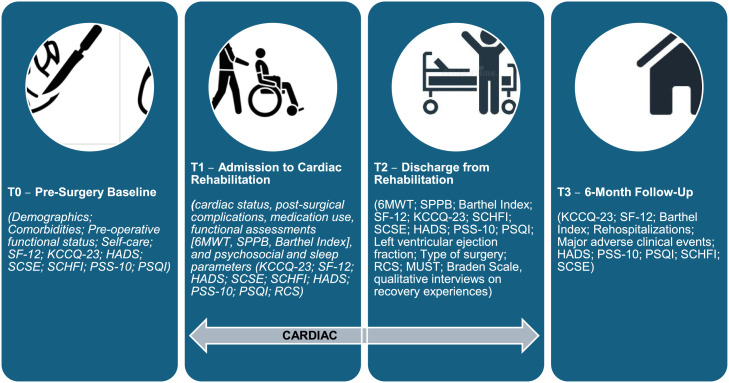
Timeline of data collection for the VALCAR(E)_QoL study. Legend: SF-12: 12-Item Short Form Health Survey; KCCQ-23: Kansas City Cardiomyopathy Questionnaire – 23 items; HADS: Hospital Anxiety and Depression Scale; SCSE: Self-Care Self-Efficacy Scale; SCHFI: Self-Care of Heart Failure Index; 6MWT: Six-Minute Walk Test; SPPB: Short Physical Performance Battery; PSS-10: Perceived Stress Scale – 10 items; PSQI: Pittsburgh Sleep Quality Index; RCS: Rehabilitation Complexity Scale; MUST: Malnutrition Universal Screening Tool.

To identify latent patient endotypes associated with KCCQ outcomes, unsupervised clustering will be performed exclusively on T0 and T1 variables to avoid circularity with T2/T3 outcomes. Mixed‑type preprocessing will use factor analysis of mixed data to preserve joint structure across continuous and categorical inputs. Clustering will be conducted using complementary methods suitable for mixed data: latent class/profile analysis and k‑prototypes with agglomerative clustering with Gower distance included as a robustness check. The optimal number of clusters/classes will be selected using BIC/ICL (model‑based methods), the gap statistic, and generalized silhouette width, supported by bootstrap stability indices (average Jaccard similarity). A minimum cluster prevalence of ≥ 5–7 % will be required to ensure interpretability. After finalizing the clustering solution and keeping it blinded to outcomes, associations between cluster membership and KCCQ‑23 at T2 and T3 will be tested using linear models with cluster as a fixed factor. Results will be reported adjusted marginal means (95 % CIs), standardized mean differences, and partial η² with related corrections for multiple comparisons when appropriate. Finally, to appraise utility for prediction, cluster membership will be added to the T3 supervised models. Incremental performance will be evaluated against models without cluster information by changes in cross‑validated R², RMSE, and calibration metrics. All unsupervised analyses will be explicitly exploratory and interpreted as phenotype characterization rather than causal inference (Fig. 1).

#### Secondary outcomes

For secondary outcomes, the same analytical strategy and sequential steps described for the primary outcome will be applied, with appropriate adaptations according to the outcome type (continuous or categorical). Regarding the analysis of the findings from the semi-structured interview, data analysis will be conducted following the criteria of qualitative narrative research. All interviews will be fully transcribed by the principal investigator to ensure fidelity to the original content. The analysis will include in-depth reading by members of the research team and the identification of common themes and recurring narrative sequences. To minimize the influence of preconceptions and researcher biases, the "bracketing" method will be applied, a reflective process that enables researchers to suspend prior assumptions and focus exclusively on participants’ lived experiences. As defined by Gearing [38], bracketing is a "scientific process in which the researcher suspends or puts on hold his or her presuppositions, prejudices, assumptions, theories, or previous experiences in order to observe and describe the phenomenon" [38,39]. During the analytical phase, relevant excerpts will be highlighted and segmented into units of meaning that capture key aspects of the phenomenon under study. These units will then be transformed into core main themes and further refined into subthemes through the method of free imaginative variation. The analysis will be conducted at multiple interpretative levels to explore the nuanced dimensions of participants’ narratives [40]. To ensure methodological robustness, the prolonged involvement of the research team, reflexive documentation, and the use of field notes and analytic memos to enrich data interpretation and ensure transparency.

## Ethical considerations

The investigators will ensure that the study is conducted in full compliance with international regulations and their national implementations regarding clinical research, as well as with the ethical principles outlined in the Declaration of Helsinki, to ensure maximum protection for all participants. The principal investigator is responsible for ensuring that the study is conducted in accordance with this protocol and with *Good Clinical Practice* (GCP) guidelines. The study promoter will ensure the protection of participants’ sensitive personal data, both clinical and nonclinical, in accordance with Italian data protection legislation (Legislative Decree 196/2003) and subsequent amendments. The study will be conducted in compliance with the national regulations on good clinical practice (Ministerial Decree 27 April 1992 as amended by the Ministerial Decree 15 July 1997 and Legislative Decree 211/2003).

## Ethics committee approval

Ethical approval for this study was granted by the *Ethics Committee of Regione Toscana – Area Vasta Centro* (approval no 28,685_oss, issued on 2 September 2025).

## Patient information and consent

All patients will be invited to participate voluntarily after receiving a detailed explanation of the study procedures and objectives. Participants will have the opportunity to ask questions and receive clear and comprehensive answers before providing consent. They will be informed that the participation is entirely voluntary and that they may withdraw from the study at any time without providing justification and without any impact on their ongoing medical care.

## Data quality and database management

All study data will be pseudonymized and entered into a secure REDCap database hosted by the Don Carlo Gnocchi Foundation [31]. Each participant will receive a unique study ID, while identifiable information will be stored separately in a password-protected log accessible only to authorized personnel. Data collectors will receive standardized training, and source data will be entered into REDCap using electronic forms with built-in range checks and validation rules. The principal investigator or data manager will regularly review the database for completeness and consistency, resolving discrepancies by checking source documents. All changes will be tracked through the REDCap audit trail. Paper documents will be stored in locked cabinets, and electronic data will be protected through user-specific access rights. A double data check will be performed before each record is locked for analysis. The principal investigator will act as data custodian and ensure confidentiality of all de-identified datasets.

## Data confidentiality and privacy

Participant privacy and data confidentiality are given the highest priority in this study. All data collected will be linked to participants only through a unique study ID; no directly identifying personal information (such as name, exact date of birth, or address) will be entered into the study database. The link between participant identity and the study ID (the key) will be stored separately, as described in the data management section, and will remain strictly confidential. Access to this key will be restricted to the principal investigator and essential research staff, who will store it in a secure, encrypted file. The handling of personal data complies with Italian privacy legislation and the European Union’s *General Data Protection Regulation* (EU GDPR, Regulation 2016/679). Participants will be fully informed about how their data will be used, stored, and protected, and that all data will be used exclusively for the aims of this scientific research. In any publications or presentations, only aggregated data or anonymized quotes from interviews will be presented to ensure that individual participants cannot be identified. All study-related documents will be stored for the period required by local regulations (typically 5–10 years after study completion) and then disposed of securely.

## Data publication

Study findings will be disseminated through presentations at scientific conferences and publications in peer-reviewed journals. Any subgroup analyses will require prior approval from the research team. The project adheres to an open-science philosophy, aiming for full transparency through open-access publications, publicly available anonymized datasets, and shared analytic code developed during the project.

## Protocol validation and limitations

This study will have several limitations. First, it will be a single-center observational design, which may limit generalizability. Second, the reliance on self-reported data measures will introduce potential response and recall bias. Participant dropout may also lead to incomplete longitudinal data, affecting representativeness. Finally, although the qualitative component will provide valuable contextual insights, it may not fully capture the heterogeneity of patient experiences.

## CRediT author statement

**Hamilton Dollaku, Piergiuseppe Liuzzi, and Camilla Elena Magi** contributed to the study conceptualization, design of the methodology, data collection, data curation, and statistical analysis, and drafted the original version of the manuscript. **Mariachiara Figura, Francesco Limonti, Biagio Nicolosi, and Duccio Frangi** were responsible for data acquisition, verification, and validation, and contributed to the critical revision of the manuscript. **Ercole Vellone, Paolo Iovino, Andrea Mannini, and Claudio Macchi** supervised the overall project, coordinated administrative and funding activities, and revised the manuscript for important intellectual content. All authors read and approved the final version of the manuscript.

## Declaration of competing interest

The authors declare that they have no known competing financial interests or personal relationships that could have appeared to influence the work reported in this paper.

## Data Availability

No data was used for the research described in the article.
